# Tear miRNAs Identified in a Murine Model of Sjögren’s Syndrome as Potential Diagnostic Biomarkers and Indicators of Disease Mechanism

**DOI:** 10.3389/fimmu.2022.833254

**Published:** 2022-03-04

**Authors:** Shruti Singh Kakan, Maria C. Edman, Alexander Yao, Curtis T. Okamoto, Annie Nguyen, Brooke E. Hjelm, Sarah F. Hamm-Alvarez

**Affiliations:** ^1^ Department of Pharmacology and Pharmaceutical Sciences, School of Pharmacy, University of Southern California, Los Angeles, CA, United States; ^2^ Department of Ophthalmology, Roski Eye Institute, Keck School of Medicine, University of Southern California, Los Angeles, CA, United States; ^3^ Department of Translational Genomics, Keck School of Medicine, University of Southern California, Los Angeles, CA, United States

**Keywords:** miRNA, NOD mouse, autoimmune dacryoadenitis, Sjögren’s syndrome, next gen sequencing, autoimmune diseases, dry eye

## Abstract

**Objective:**

The tear miRNAome of the male NOD mouse, a model of ocular symptoms of Sjögren’s syndrome (SS), was analyzed to identify unique miRNAs.

**Methods:**

Male NOD mice, aged 12-14 weeks, were used to identify tear miRNAs associated with development of autoimmune dacryoadenitis. Age- and sex-matched male BALB/c mice served as healthy controls while age-matched female NOD mice that do not develop the autoimmune dacryoadenitis characteristic of SS were used as additional controls. Total RNA was isolated from stimulated tears pooled from 5 mice per sample and tear miRNAs were sequenced and analyzed. Putative miRNA hits were validated in additional mouse cohorts as well as in tears of SS patients versus patients with another form of dry eye disease, meibomian gland disease (MGD) using qRT-PCR. The pathways influenced by the validated hits were identified using Ingenuity Pathway Analysis.

**Results:**

In comparison to tears from both healthy (male BALB/c) and additional control (female NOD) mice, initial analy1sis identified 7 upregulated and 7 downregulated miRNAs in male NOD mouse tears. Of these, 8 were validated by RT-qPCR in tears from additional mouse cohorts. miRNAs previously implicated in SS pathology included mmu-miR-146a/b-5p, which were significantly downregulated, as well as mmu-miR-150-5p and mmu-miR-181a-5p, which were upregulated in male NOD mouse tears. All other validated hits including the upregulated miR-181b-5p and mmu-miR-203-3p, as well as the downregulated mmu-miR-322-5p and mmu-miR-503-5p, represent novel putative indicators of autoimmune dacryoadenitis in SS. When compared to tears from patients with MGD, miRNAs hsa-miR-203a-3p, hsa-miR-181a-5p and hsa-miR-181b-5p were also significantly increased in tears of SS patients.

**Conclusions:**

A panel of differentially expressed miRNAs were identified in tears of male NOD mice, with some preliminary validation in SS patients, including some never previously linked to SS. These may have potential utility as indicators of ocular symptoms of SS; evaluation of the pathways influenced by these dysregulated miRNAs may also provide further insights into SS pathogenesis.

## Introduction

Sjögren’s Syndrome (SS) is a chronic, progressive autoimmune disease that affects ~1% of the population (1) and causes inflammation of moisture-producing glands including lacrimal glands (LG) and salivary glands (SG), leading to dry eye and dry mouth (1). SS also causes systemic disease including inflammation in skin, lungs, kidneys, and the nervous system resulting in dryness of the skin, nose and vagina, debilitating muscle and joint pain, fatigue, and chronic cough (2). Among all autoimmune diseases, SS patients have the highest incidence of malignant lymphoma (3, 4). Diagnosis of SS is challenging because the current diagnostic criteria involve multiple subjective and analytical tests including a blood draw and an invasive minor SG biopsy (5, 6). Additionally, symptoms of SS overlap with those of other autoimmune diseases such as Rheumatoid Arthritis (RA) and Systemic Lupus Erythematosus (SLE) and other dry eye diseases such as Meibomian gland disease (MGD), leading to delays in diagnosis or misdiagnosis (7). Thus, it can take several years before the disease is confirmed, during which time infiltrating immune cells may further damage exocrine glands and sustain debilitating ocular and oral cavity symptoms. SS can also lead to other ocular complications such as uveitis and optic neuritis (8, 9). Although it is the second most common autoimmune disorder in the United States (10), SS receives far less attention for research and therapeutic development than does RA or SLE. With growing prevalence, there is a need for more specific diagnostic tests for SS to prevent irreversible tissue and/or organ damage and to improve the health and vision-related quality of life for SS patients.

An ideal diagnostic tear biomarker for SS would be chemically stable and able to: 1) detect SS with high sensitivity and specificity (11); 2) distinguish SS from other autoimmune and dry eye diseases; 3) be collected relatively non-invasively (11); and 4) be processed inexpensively in a straightforward manner (11, 12). With these characteristics in mind, we investigated a type of short non-coding RNAs called microRNA (miRNAs) that are 18-26 nucleotides long. miRNAs circulate in the body either packaged inside secreted vesicles or bound to RNA-binding proteins and are therefore highly stable and protected from RNAse degradation. miRNAs are responsible for transcriptional regulation of nearly 60% of all mammalian messenger RNA (mRNAs) (13), and dysregulation of miRNAs has been implicated in diseases such as cancer and neurodegenerative disease (14, 15). Moreover, miRNAs and their mRNA targets have co-evolved and are highly evolutionarily conserved in mammals (13), allowing for studies in model organisms to be directly extrapolated to humans. This feature is critical for SS research, as mammalian model organisms allow investigation of the earliest stages of glandular inflammation, which is difficult to do in patients due to delays in SS diagnosis

While SS causes systemic symptoms, its hallmark manifestations are inflammation of LG and SG. Although other biofluids and their components, i.e., plasma/serum, peripheral blood mononuclear cells (PBMC) and saliva, have been extensively investigated as sources of protein and RNA based biomarkers, there are limited reports of the assessment of tears for the presence of select miRNAs in SS (16) and none using comprehensive Next Generation Sequencing (NGS) analysis. Tears are highly enriched in miRNAs compared to other biofluids such as serum and cerebrospinal fluid (17). Moreover, tear miRNA candidates have been investigated for detection of primary open-angle glaucoma (18) and Alzheimer’s disease (15), with very high sensitivity and specificity. Tear collection is quick, atraumatic, and non-invasive. Most importantly, changes in tear composition are likely to be directly related to the health of the LG, the principal source of most of the aqueous tear components and a primary target of autoimmune exocrinopathy (19). Thus, we propose that dysregulated tear microRNA may serve as indicators of LG disease in Sjögren’s syndrome and might also provide insights into disease pathogenesis.

This discovery study utilized male NOD mice, a well-established model of the ocular symptoms of SS. These mice exhibit lymphocytic infiltration of the LG, beginning around six weeks of age and by 12-14 weeks of age, they exhibit a notable lymphocytic infiltration of LG associated with symptoms of dry eye disease (20, 21). There are no ideal murine models of SS that accurately recapitulate all of the elements of human disease, but the male NOD mouse has proven an excellent choice for study of ocular symptoms. Importantly, by 12-14 weeks of age as used in this study, male NOD mice displays multiple key characteristics of SS-like disease similar to humans including a) lymphocytic infiltration in LG resulting in autoimmune dacryoadenitis (21) b) loss of secretory function in LG (22, 23) c) development of anti-SSA/SSB and anti-muscarinic receptor type III autoantibodies (23–25); d) an increased degradation of ECM proteins and deformation of ECM structures in LG (26); and e) increased proteolytic enzymes in tears (20). Importantly, we first discovered elevated tear cathepsin S as a putative biomarker of SS-associated dry eye disease in the male NOD mice (20), a finding that was later validated in tears of female SS patients when compared to tears of healthy controls, and other dry eye and autoimmune disease patients (27). As well, male NOD mice manifest disease within a rapid time frame, unlike other transgenic or knockout models which require extended aging (28, 29).

Age- and sex-matched male BALB/C mice, which lack LG disease and are frequently used as a control for male NOD and their derivatives in studying disease development (20, 30), served as the primary healthy control. Additionally, female NOD mice do not manifest marked ocular symptoms of SS, instead exhibiting SG inflammation at a later age starting at 16 weeks. They are typically used to study autoimmune sialadenitis. At 12-14 weeks, female NODs do not exhibit lymphocytic infiltration in their LG, and have intact ocular surfaces and tear production comparable to healthy female BALB/c mice. The minimal local LG disease that they may express occurs beyond 5 months of age (31, 32). Female NOD mice share the genetic background of the male NOD mice and so, age-matched female NOD mice were used as additional controls.

**Figure 1:**
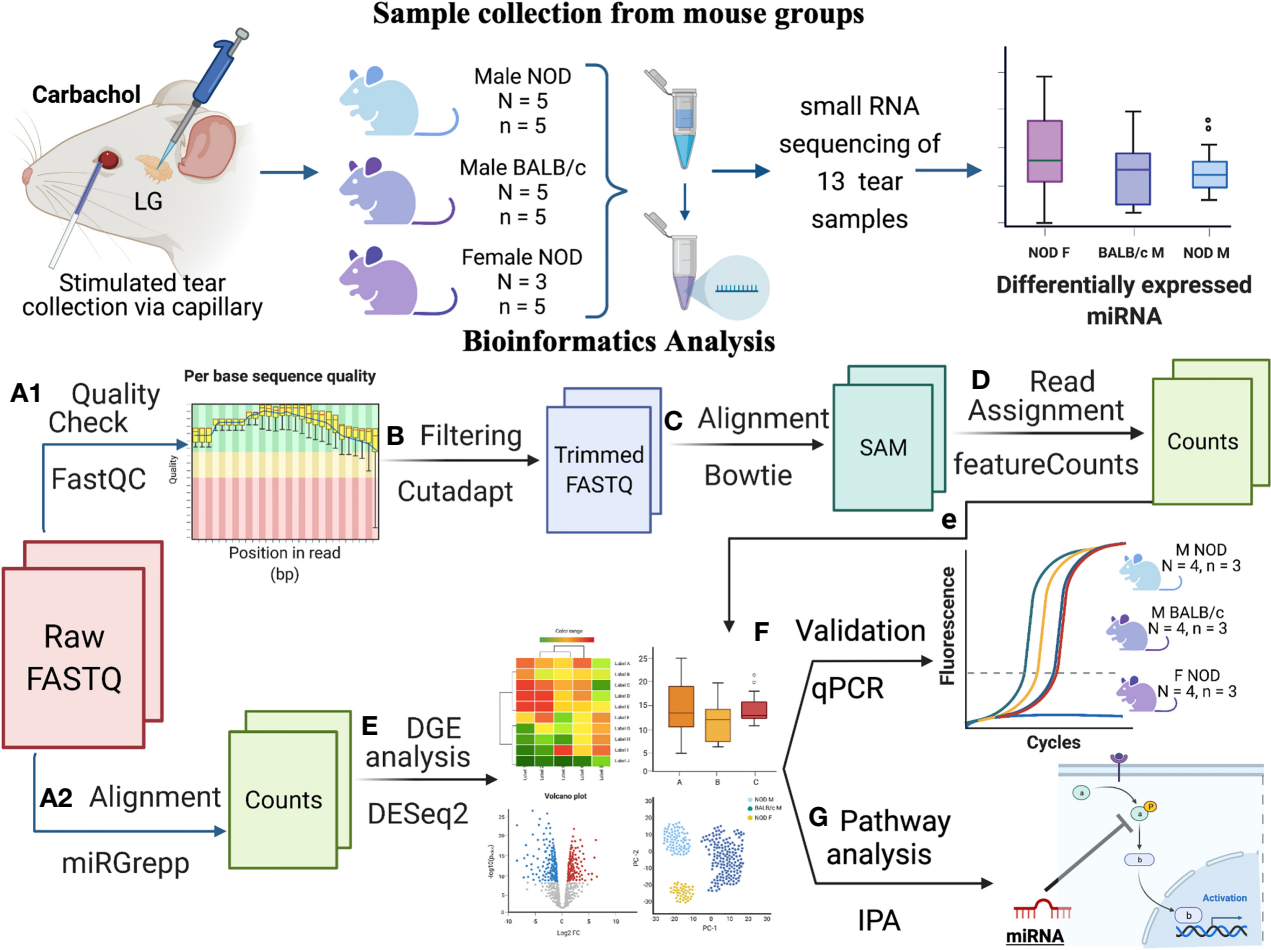
Schematic overview of experiments and data analysis. Tears were collected by stimulating both LG from each mouse with 50 μM Carbachol. Tears were pooled from n=5 mice for each sample, with N=5 samples for male NOD and BALB/c mice and N=3 for female NOD mice. In total, 13 samples were sent for RNA sequencing, and raw data was analyzed as follows: **(A1)** Quality assessment by FASTqc showed presence of 3’ adapters in the reads; **(B)** Using cutadapt (33), 3’ adapters were trimmed and reads with quality scores <20 or length <15 were removed; **(C)** Trimmed reads were aligned to mouse genome GRCm38 using Bowtie (34); and **(D)** Aligned reads were annotated using featureCounts (35). Additionally, **(A2)** raw FASTQ files were also run through our internal miRGrepp pipeline (36) and **(E)** miRNA counts were normalized, and plotted to assess the quality of the data with statistics on these read counts performed using R package, DESEq2 (37); **(F)** Shortlisted hits were validated by qPCR in a separate cohort of mice; and and **(G)** Pathway analysis of the hits was done in Ingenuity Pathway Analysis (Qiagen). Graphic created with BioRender.com.

In this study, stimulated tears were collected from each mouse cohort and pooled for isolation of total RNA, prior to conducting a comprehensive small RNA sequencing analysis (**Figure 1**). Over 500 distinct miRNAs were identified in male NOD mouse tears of which 14 were differentially expressed compared to tears from both control groups. These findings were validated in additional cohorts of mice using qRT-PCR, confirming differential expression of 8 miRNAs. In tears of 12-14 week old male NOD mice, miR-146a-5p, miR-146b-5p, miR-322-3p, and miR-503-5p were down-regulated; whereas miR-181a-5p, miR-181b-5p, miR-150-5p, and miR-203-3p were up-regulated. In a pilot study, we have also conducted a preliminary analysis of the presence and abundance of these miRNAs using qRT-PCR in tears of patients with SS-associated dry eye disease versus patients with another form of dry eye due to meibomian gland disease (MGD). MGD is associated with changes in tear lipid secretion resulting in more rapid evaporation and destabilization of the tear film (38). MGD is the most common cause of non-autoimmune evaporative dry eye (39). In contrast, dry eye in SS is primarily aqueous-deficient, resulting from autoimmune damage to the LG; however a component of evaporative dry eye may develop with time due to consequent inflammation of meibomian glands (40). Using MGD patients as a non-autoimmune dry eye control group, we assessed whether miRNA hits identified from our NOD studies could distinguish early SS disease resulting from autoimmune LG dysfunction. Three of the 8 miRNAs identified from male NOD mice versus controls were significantly upregulated in SS tears relative to MGD tears– hsa-miR-203a-3p, hsa-miR-181a-5p and hsa-miR-181b-5p. To our knowledge, this is the first comprehensive tear miRNA NGS analysis in an animal model of SS, the results of which may have important implications for improved understanding of mechanisms involved in disease as well as impact diagnosis of ocular involvement in SS patients.

## Materials and Methods

### Animals

Male NOD/ShiLtJ (001976) mice aged 12-14 weeks were used as an early-intermediate disease model of ocular manifestations of SS (20, 41). Longitudinal studies confirm that in the age range of 12-14 weeks in male NOD mice, there is no significant difference in tear production or lymphocytic infiltration in the LG (31, 42, 43). While age-matched male BALB/cJ (000665) mice served as a healthy control, female NOD/ShiLtJ mice that do not develop lymphocytic infiltration in the LG or other ocular manifestations of Sjögren’s syndrome by 14 weeks were employed as an additional, strain-specific control (31). Mice older than 14 weeks were excluded to avoid potential confounding effects of diabetes. All strains were purchased from Jackson Laboratories (Bar Harbor, Maine). All animal procedures and experiments followed protocols approved by the Institutional Animal Care and Use Committee (IACUC) at the University of Southern California (Protocol #10547) as well as the Guide for Care and Use of Laboratory animals, Eighth Edition (44).

### Human Subjects

Deidentified tear samples were obtained from the USC Dry Eye Center of Excellence tear biorepository at the USC Roski Eye Institute. Use of samples from the tear biorepository is approved under IRB protocol HS-19-01004 and meets the requirements for ‘non-human research with biospecimens previously generated/collected to be analyzed for research purposes (Data and Specimens) or secondary data analyses’. Samples obtained belonged to 6 SS and 6 age-matched meibomian gland disease (MGD) patients undergoing treatment at the LAC+USC Medical Center or Keck Medicine of USC Rheumatology clinics. The diagnosis of SS was based on the 2016 American College of Rheumatology/European League Against Rheumatism (ACR-EULAR) consensus classification criteria for SS (45) or alternatively, by referral to the attending ophthalmologist as SS patients by rheumatologists. Three of six SS patients were determined positive in ophthalmology based on the 2016 ACR-EULAR criteria. The remaining 3 patients were referred to the attending ophthalmologist as SS patients who met clinical criteria in ophthalmology for keratoconjunctivitis sicca (ocular staining score ≥ 5 and/or Schirmer’s test ≤ 5 mm in 5 min in at least 1 eye). MGD-dominant dry eye patients served as controls since lipid secretion required to control evaporation and maintain a normal tear film is abnormal in these patients, leading to evaporative dry eye. This contrasts to SS-related dry eye where aqueous deficient dry eye results from the lack of aqueous tear secretion by the LG due to inflammatory changes, although, evaporative and aqueous deficient dry eye are not mutually exclusive. The diagnosis of MGD was made clinically by the ophthalmologist based on presence of obstruction of meibomian glands due to increased viscosity of meibum or hyperkeratinization of the glands and leading to decreased secretion of meibum (38). This decrease can affect tear film stability (measured by tear break-up time, lipid layer thickness, and/or tear osmolarity) (38). Written informed consent was obtained from each patient and their samples were deidentified prior to depositing in the biorepository.

### Tissue and Tear Collection

Tears were analyzed from two separate groups of mice, a discovery cohort used for library preparation and NGS sequencing, and a validation cohort, used for RNA isolation and qRT-PCR analysis to validate putative hits obtained by NGS. Stimulated tears were collected from mice as described previously (20). Briefly, mice were anesthetized with 20 mg/kg of Ketamine and 2 mg/kg of Xylazine and placed on heating pads. An incision was made in the skin above the LG, and the exposed gland was stimulated with 3 μL of 50 μM carbachol (Alfa Aesar, Haverhill, MA); carbachol is a muscarinic and nicotinic agonist that efficiently evokes stimulated secretion of tear proteins by the lacrimal gland through activation of M3 muscarinic receptors (46). A minimum of two mice producing a total of at least 6 uL of tears was required to isolate sufficient RNA detectable by nanodrop or TapeStation. Tears were collected using 2 μL sized Microcaps glass capillaries (Drummond Scientific, Broomall, PA) for 5 min after topical stimulation. This process was repeated twice for a total of 15 min of stimulated collection, after which the mice, still under anesthesia were euthanized by cervical dislocation. One LG (either left or right) was selected at random for RNA isolation and the remaining LG was processed for histology using Hematoxylin and Eosin (H & E) staining. Tears were pooled from 5 mice for a single sample intended for Next Generation Sequencing (NGS) (to allow collection of sufficient RNA for NGS library preparation), with 5 samples each for male NOD and BALB/c mice and 3 samples for female NOD mice. As female mice represented an additional control beyond the age- and sex-matched male BALB/c, we used a smaller number of total samples of pooled tears for this group in the discovery cohort only; the numbers used for validation were equivalent for all strains.

For RT-qPCR validation experiments, tears were similarly collected; however, tears comprising one sample were pooled from 3 mice from each mouse group. Four pooled samples per group were analyzed. Cost constraints were the primary reason for the choice of the sample size for discovery and validation cohorts. The number of mice used per sample to pool tear RNA was determined by the need to exceed the minimum amount of RNA required for NGS library preparation or RT-qPCR. We used 5 mice per group to target a goal of 50 ng per sample for NGS library preparation. For the validation cohorts, all samples included pooled tears from three mice, as qRT-PCR required less than 15 ng of total RNA.

Human tear samples were collected from filter paper used for Schirmer’s test. For the test, filter paper strips were placed in the inferior fornix of each eye after topical application of 0.5% proparacaine hydrochloride ophthalmic solution. The strips were removed after five min and Schirmer’s Score (i.e., length of tear saturation in mm) was recorded (27). Strips from each of left and right eyes were placed in separate deidentified tubes and stored at -80°C.

### LG Histology and Quantification of Lymphocytic Infiltration

LGs were placed in cassettes which were then fixed in 10% neutral buffered formalin solution for at least 24 h and transferred to 70% ethanol. Tissues were embedded in paraffin, and longitudinal cross-sections of 5-6 µm thickness were cut at various depths into the LG. Each tissue section was stained with H&E dye and images were acquired with an AxioScope 5 with Axiocam 305 microscope (Carl Zeiss AG, Jena, Germany). Infiltrates were quantified using Image J (47) to calculate the total area of the LG and the area occupied by infiltrating lymphocytes. The percentage infiltration was calculated by dividing the area occupied by foci divided by the total area of the cross-section multiplied by 100. The percentage of infiltration at three depths within the LG in each mouse was averaged to reflect the total amount of lymphocytic infiltration/gland.

### RNA Isolation and Quality Control

5 µL of β-mercaptoethanol (BME) was added to each pooled mouse tear sample to prevent degradation by RNAase. Addition of BME to tears improved RNA quality as indicated by improvement of Agilent Tapestation traces and RNA integrity Number (RIN) (data not shown). Total RNA was isolated using the miRNEasy total RNA isolation kit (Qiagen) following the manufacturer’s guide, with a slight change in the final step. RNA was eluted from the dried column by adding 14 µL of RNAse free water and eluting twice for a total elution volume of ~28 µL. RNA was stored at -80°C prior to library preparation. RNA quality and concentration was assessed in house using TapeStation with a High Sensitivity RNA ScreenTape (5067-5579, Agilent) to ensure the RNA was of high integrity and of sufficient quantity for NGS library preparation. Samples with RIN scores < 2 or RNA amount < 10 ng were excluded from sequencing.

Human tear samples were stored on Schirmer’s strips at -80°C. Tears were eluted from Schirmer’s strips in 0.9 mL of Qiazol reagent (Qiagen), combined in a single tube and total RNA was isolated using the miRNeasy Serum/plasma total RNA isolation kit (Qiagen). RNA concentration, yield and quality were assessed using TapeStation. Samples with RNA Integrity Numbers (RIN) 3 or higher were used for downstream cDNA synthesis and qPCR analysis.

### Library Preparation, NGS, and Bioinformatics Analysis

Library preparation and NGS was outsourced to Qiagen where the RNA quality was again assessed using Nanodrop and TapeStation prior to library preparation. The order of samples for library preparation and NGS was not known to the authors. The small RNA library was prepared using the QIAseq miRNA library kit (331502, Qiagen), specifically optimized for very low input small RNA, following the manufacturer’s guidelines. Small RNA sequencing was conducted on an Illumina Nextseq 500/550 high output flow cell with 75 cycles in single end configuration with an average sequencing depth of 17 to 18 million reads/sample (expected to be sufficient to provide >1 million mapped miRNA reads per sample to allow sufficient power to detect differentially expressed miRNA in accordance with exRNA consortium guidelines).

Read quality of the FASTQ files from all samples were assessed using FASTQC; adapter and quality trimming was done with Cutadapt v2.0. Trimmed reads were aligned to mouse whole genome GENCODE GRCm38 using Bowtie and quantified using featureCounts. Raw reads were also aligned to miRbase v22.0 as described previously (36) using our in-house aligner miRGrep (36). miRNA read counts filtering, data normalization, and differential gene expression analysis was done using DESEq2 in RStudio with no data points excluded in the analysis. (A full list of software packages, R Code and program parameters can be found in **Figure 1**).

### RT-qPCR Validation

Validation of miRNA hits utilized RT-qPCR analysis of tear miRNA isolated from separate cohorts of mice and dry eye patients using Qiagen’s Custom PCR Panels. 13 miRNAs were tested. Due to cost and other constraints in designing the custom PCR panel to validate hits, miR-322-3p was not included. We reasoned that if the 5p counterpart evaluated in the panel was confirmed as differentially expressed, it would be reasonable to expect that the 3p counterpart might also be differentially expressed since they are the products of the same precursor miRNA, miR-322. cDNA was prepared from 10 ng of total RNA using miRCURY LNA-enhanced cDNA synthesis kit (Qiagen) followed by PCR with 100 ng of cDNA using custom made PCR panels (Qiagen) which were pre-coated with test and house-keeping miRNA primers. Real time PCR was done in triplicates for 40 cycles on a QuantStudio 6000 flex instrument (Applied Biosystems, Foster City, CA, USA). Raw Ct values were first normalized using the inter-plate calibrator UniSp3, followed by normalization to internal controls, miR-25-3p and miR-16-5p. Statistics were performed on ΔCt values in GraphPad prism v7.0. Results were reported with respect to controls using the ΔΔCt method.

### Ingenuity Pathway Analysis of miRNA Hits

miRNA hits validated by qPCR were uploaded to Ingenuity Pathway Analysis (IPA) with fold-change and p-values calculated from DESeq2. Using the ‘microRNA target filter’ module of IPA, a list of mRNA targets sourced from TargetScan was obtained. IPA recognized seven microRNA hits and reported a list of 3991 mRNA targets. This list was shortlisted by a) confidence (experimentally validated, predicted high) to 1100 mRNA targets, and b)species (restricted to mammals only– human, mouse, and rat), from which, a network map was generated. Using the build and connect features of the main ‘pathways’ module, mRNA targets involved in immunological and inflammatory diseases, inflammatory responses, and cancer were shortlisted. Pathways, disease categories and functions over-represented by the miRNA and their mRNA targets were obtained using the overlay feature of the ‘pathways’ module in decreasing order of probability. From here, pathways most significantly over-represented were evaluated to gain further insights into the potential role of the dysregulated miRNA hits in SS.

## Results

### Histological Analysis of LG Lymphocytic Infiltration

**Figure 2:**
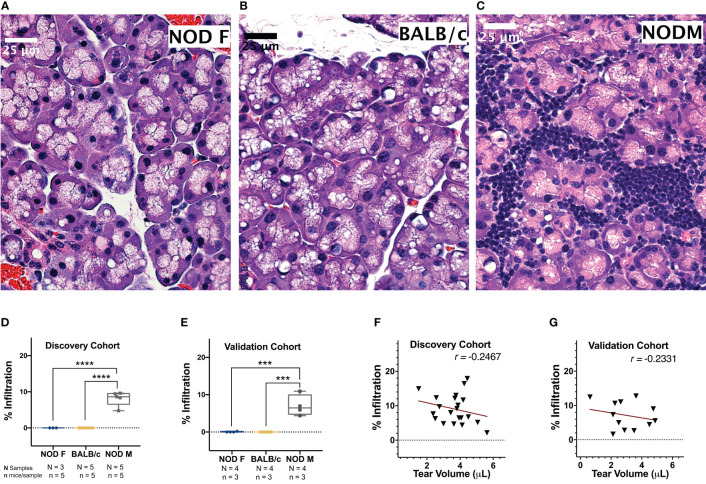
Representative H & E-stained images of LG from 12 -14-week-old female NOD, male BALB/c or male NOD mice. No lymphocytic infiltration is observed in LG from **(A)** female NOD mice or **(B)** male BALB/c mice. Marked lymphocytic infiltration is observed in LG from **(C)** male NOD mice. Scalebar, 25 μm. The percentage of total area of infiltrating lymphocytes in LG calculated in both the **(D)** discovery cohort and **(E)** validation cohort of age-matched female NOD mice (blue), male BALB/c mice (yellow) and male NOD mice (grey). Points represent the percentage lymphocytic infiltration for one LG per mice. Data are plotted as a boxplot with whiskers showing range. (***p < 10^-3^, ****p < 10^-4^, One-Way ANOVA). The correlation between percentage lymphocytic infiltration and tear production for male NOD mice was calculated in both **(F)** discovery and **(G)** validation cohorts. Pearson’s correlation coefficient (*r*) is shown. Points in D-G represent individual mouse LG **(D, E)** or mouse tears **(F, G)** with N=25 for male NOD and BALB/c and N=15 for female NOD for discovery cohorts and N=12 for all mouse groups in the validation cohort.

To determine the percentage of lymphocytic infiltration in mouse LG, H&E-stained images of LG sections from the mice used for the initial tear collection were acquired and analyzed. Neither the LG of female NOD mice (**Figure 2A**) or healthy male BALB/C mice (**Figure 2B**) at 12-14 weeks of age showed any lymphocytic infiltration, as expected (31). The male NOD LG had notable lymphocytic infiltration from 12-14 weeks of age (**Figure 2C**), confirming establishment of LG disease associated with SS. The percentage infiltration of LG from the male NOD mice was thus significantly higher than both the male BALB/c and female NOD mice in both discovery (**Figure 2D**) and validation (**Figure 2E**) cohorts.

Tear production in male NOD mice was significantly lower than male BALB/c mice in both the discovery (p < 10^-4^) and validation (p < 10^-4^) cohorts (**Supplemental Figure S1A**). Tear production in male NOD was not significantly different from female NOD mice in the discovery cohort but was significantly lower in the validation (p < 10^-3^) cohort. This is likely due to the 1.25-fold lesser body weight and 1.75-fold smaller size of female mouse LG relative to their male counterparts (32), as tear volumes for female NOD and female BALB/c mice were comparable (**Supplemental Figure S1B**). Tear production in male NOD mice was weakly inversely correlated to the extent of lymphocytic infiltration in the LG in mice from both the discovery cohort (**Figure 2F**) (*r* = -0.2467, Pearson’s correlation coefficient) and the validation cohort (**Figure 2G**) (*r* = -0.2331, Pearson’s correlation coefficient). It has been previously published that lymphocytic infiltration is only one of several determinants of reduced tear flow (48, 49).

### Male NOD Mice Have Differential Tear Production, but Not Total Protein or RNA Yields

As the LG is the predominant source of aqueous tear components, changes in miRNA composition of tears may be indicative of LG disease. We have established previously that male NOD mice have decreased tear production, similarly to SS patients (20, 27). However, their tear protein concentration when normalized to tear volume is not affected (20). Similarly, we did not observe a significant difference in the RNA tear concentration normalized to tear volume in the male NOD mice as compared to male BALB/c and female NOD mice (**Supplemental Figure S1C**). There were also no significant differences in the RNA concentration or quality, as indicated by the RNA integrity number (RIN) of samples in each group (**Supplemental Figure S1D**). Assessing the total number of reads aligned to miRbase v22, the counts per million miRNA or CPM miRNA normalized to RNA amount per unit tear volume, showed no significant difference across the three groups (**Supplemental Figure S1E**).

### Male NOD Mice Tears From the Discovery Cohort Exhibit Differentially Expressed miRNA

**Figure 3:**
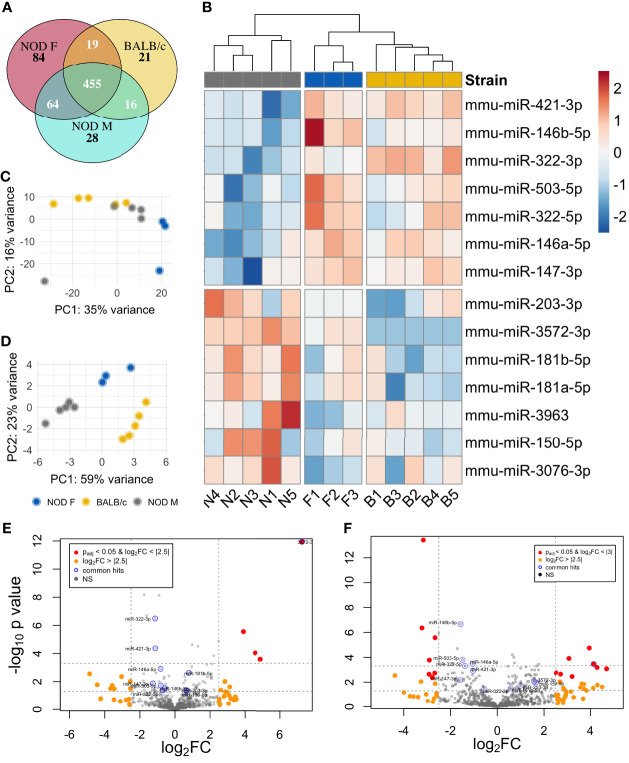
Differential miRNA expression analysis of NOD mouse tears. **(A)** Venn diagram showing the overlap of miRNA in the three strains. miRNA that had normalized read counts > 0 in at least 4 out of 5 samples of NOD and BALB/c and 2 out of 3 samples for the female NOD were included for calculations. **(B)** Heatmap of 14 miRNA that are differentially expressed in male NOD mouse tears as compared to tears of male BALB/c and female NOD. Principal component analysis (PCA) plot of the **(C)** complete miRNA data and **(D)** top miRNA hits, characterizes the trends exhibited by the expression profiles of the 3 strains. Each dot represents a sample, and each color represents the type of the sample. Volcano plot of differentially expressed miRNA in NOD mouse tears as compared to **(E)** that of male BALB/c and **(F)** female NOD.

We detected 563 distinct miRNAs in male NOD mouse tears, 511 miRNAs in the BALB/c mouse tears, and 622 miRNAs in the female NOD mouse tears (**Figure 3A**). About 455 tear miRNAs were common to all three strains while 28 and 21 miRNAs were unique to male NOD and male BALB/c mouse strains, respectively. Female NOD mouse tears had the greatest number of distinct miRNAs at 84, which is likely a sex effect. Female NODs also had more tear miRNA in common with male NOD (64) than with male BALB/c (19), which may be attributable to a strain effect. Both sex and strain were used as co-variates in the downstream statistical analysis.

**Figure 4:**
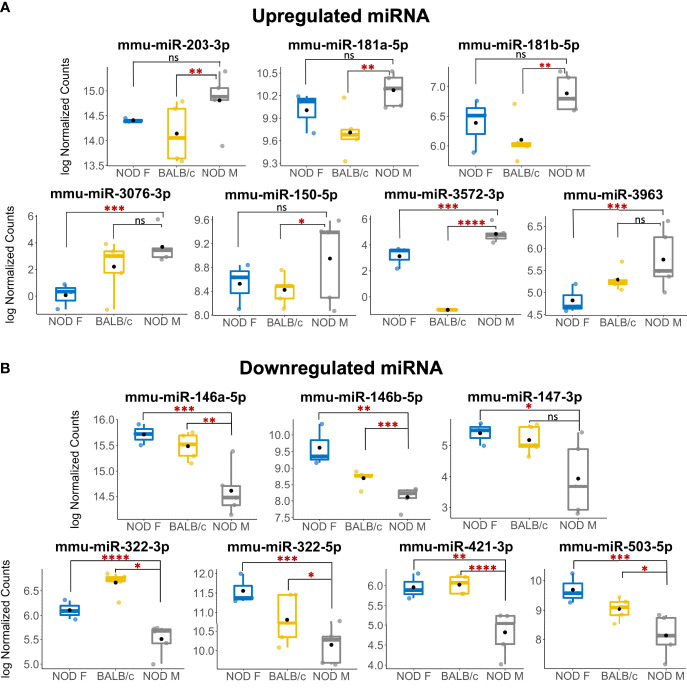
Multiple miRNAs are differentially expressed in tears of diseased male NOD mice. **(A)** 7 miRNAs were identified as upregulated in tears of 12-14 week male NOD mice while **(B)** 7 more miRNAs were found to be downregulated in tears of 12-14 week male NOD mice as compared to both age-matched female NOD and male BALB/c mice. Log10 Normalized miRNA counts as calculated by DESeq2 are plotted for each strain. N=5 samples for male NOD and BALB/c, and N=3 samples for female NOD mice; n=5 mice per sample for each strain. miRNA were considered differentially expressed if the fold change trended in the same direction for NOD M vs BALB/c and NOD M vs NOD F; had a mean expression value of 10 reads or higher and had a significant unadjusted p value in at least one of the two comparisons. (*p < 0.05; **p < 0.01, ***p < 0.001, ****p < 0.0001, DESeq2; ns, not significant).

A miRNA was considered as a ‘hit’ if it had 1) a normalized base mean expression of at least 10 reads, 2) was up or downregulated in male NOD tears in the same direction when compared to both control samples from male BALB/c and female NOD tears, and 3) resulted in a significant (p<0.05), unadjusted p-value in at least one of the two comparisons. The unadjusted p-values (**Table 1**) were used for initial selection of differentially regulated miRNAs, which were further validated by qPCR (**Table 2**). With these criteria, using DESEq2, 14 miRNAs were found to be differentially expressed (**Table 1**). Seven miRNAs (miR-181a-5p, miR-181b-5p miR-203-3p, miR-150-5p, miR-3076-3p, miR-3963, miR-3572-3p) were upregulated (**Figure 4A**), while another 7 miRNAs (miR-146a/b-5p, miR-147-3p, miR-322-3p, miR-322-5p, miR-421-3p, miR-503-5p) were downregulated in tears of male NOD mice (**Figure 4B**).

**Table 1:** Differentially expressed miRNA in male NOD mice tears as compared to those in tears of male BALB/c and female NOD mice.

miRNA	Mean Expression	Log2 Fold Change			p_value_	
		NOD M vs. NOD F		NOD M vs. BALB/c M	NOD M vs. NOD F	NOD M vs. BALB/c M
mmu-miR-203-3p	23892.76	0.656		0.477	0.1937	**0.0389**
mmu-miR-181b-5p	92.48	0.779		0.432	0.1569	**2.411 x 10^-3^**
mmu-miR-181a-5p	1042.03	0.547		0.258	0.2634	**4.470 x 10^-3^**
mmu-miR-3076-3p	10.00	1.269		4.143	**3.810 x 10^-4^**	0.0919
mmu-miR-150-5p	425.41	0.639		0.510	0.1713	**0.0473**
mmu-miR-3572-3p	14.00	7.245		1.583	**9.558 x 10^-3^**	**1.270 x 10^-12^**
mmu-miR-3963	43.81	0.595		0.999	**0.0182**	0.0872
mmu-miR-146a-5p	40196.68	-1.045		-0.826	**3.631 x 10^-4^**	**1.141 x 10^-3^**
mmu-miR-146b-5p	461.41	-1.560		-0.578	**2.406 x 10^-7^**	**0.0277**
mmu-miR-147-3p	27.53	-1.583		-1.258	**7.051 x 10^-3^**	**0.0134**
mmu-miR-322-3p	72.25	-0.544		-1.136	**0.0423**	**3.526 x 10^-7^**
mmu-miR-322-5p	1907.54	-1.376		-0.707	**4.558 x 10^-4^**	**0.0409**
mmu-miR-421-3p	50.43	-1.028		-1.125	**1.450 x 10^-3^**	**4.195 x 10^-5^**
mmu-miR-503-5p	520.23	-1.485		-0.812	**1.604 x 10^-4^**	**0.0185**

p-values and log2FoldChanges were calculated using DESeq2. Significant unadjusted p-values are in bold.

**Table 2:** qPCR validation of differentially expressed miRNA in male NOD mice tears.

miRNA	Normalized to miR-25-3p				Normalized to miR-93-5p			
	*p* value^*^		Fold Change^+^		*p* value^*^		Fold Change^+^	
	NOD M v NOD F	NOD M v BALB/c M	NOD M v NOD F	NOD M v BALB/c M	NOD M v NOD F	NOD M v BALB/c M	NOD M v NOD F	NOD M v BALB/c M
mmu-miR-181a-5p	**<0.0001**	**<0.0001**	2.253	2.058	**0.0074**	**0.0074**	2.384	1.711
mmu-miR-146a-5p	**0.0014**	**<0.0001**	-1.451	-2.441	**<0.0001**	**<0.0001**	-2.632	-3.229
mmu-miR-203-3p	**<0.0001**	**<0.0001**	2.747	1.846	**0.0111**	0.068	4.761	1.525
mmu-miR-146b-5p	0.4496	**<0.0001**	1.212	-1.821	**0.0063**	**<0.0001**	-1.667	-2.571
mmu-miR-150-5p	**<0.0001**	**<0.0001**	3.845	2.317	**0.0003**	**0.0042**	3.886	3.877
mmu-miR-322-3p	0.1580	**0.0137**	1.065	-1.806	**0.0062**	**0.0012**	-3.043	-2.978
mmu-miR-181b-5p	**0.0041**	**<0.0001**	1.939	2.238	**0.0251**	**0.0097**	3.671	3.423
mmu-miR-3963^‡^	**<0.0001**	**0.0163**	-3.386	1.380	**<0.0001**	0.477	-23.002	1.029
mmu-miR-3572-3p^†^	**0.0022**	0.5291	-1.372	1.432	**<0.0001**	0.8333	-9.319	-1.925
mmu-miR-503-5p	0.7268	0.1355	2.037	0.658	0.601	**0.0102**	2.863	1.976
mmu-miR-3076-3p	–	0.4914	1.062	1.772	–	0.0677	–	1.835
mmu-miR-421-3p	0.6508	0.9841	1.184	1.026	0.9091	0.9727	-2.441	-1.252

^*^Between group p values calculated using ΔCt values normalized to miR-25-3p and miR-93-5p, using Kruskal-Wallis ANOVA with Tukey’s multiple correction in Graph-Pad Prism. Significant p values are shown in bold; ^+^Fold changes reported are averaged ratio of ΔCt for male NOD to that of male BALB/c or female NOD; ^†^ and ^‡^Not considered a validated hit due to reversal in direction of FC between the two comparisons. ^‡^Variation is largely driven by female NOD mice.

Unsupervised hierarchical clustering analysis (shown as heatmaps) of differentially expressed miRNA clustered female NOD with BALB/c separately from male NODs (**Figure 3B**). Sample level variation is illustrated in the heatmaps. Principal component analysis (PCA) of the data shows considerable overlap between male NOD and BALB/c when comparing all miRNAs, while the female NOD samples cluster away from the males. PC1 accounts for 35% of the variation in the data, likely attributable to sex (**Figure 3C**). When comparing the top miRNA hits, however (**Figure 3D**), there do not appear to be any outliers and samples within a group correlate well. PC1 accounts for 59% of the variation and appears to show a strain effect with controls clustering close to each other and away from male NODs. PC2 accounts for 23% of the variation and shows a sex effect. Volcano plots comparing tear miRNA expression show greater variability between male and female NOD mice (**Figure 3F**) and less in male NOD and BALB/c mice tears (**Figure 3E**).

### qPCR Confirms Differential Expression of Multiple Identified miRNAs in Tears From an Additional, Validation, Mouse Cohort

**Figure 5:**
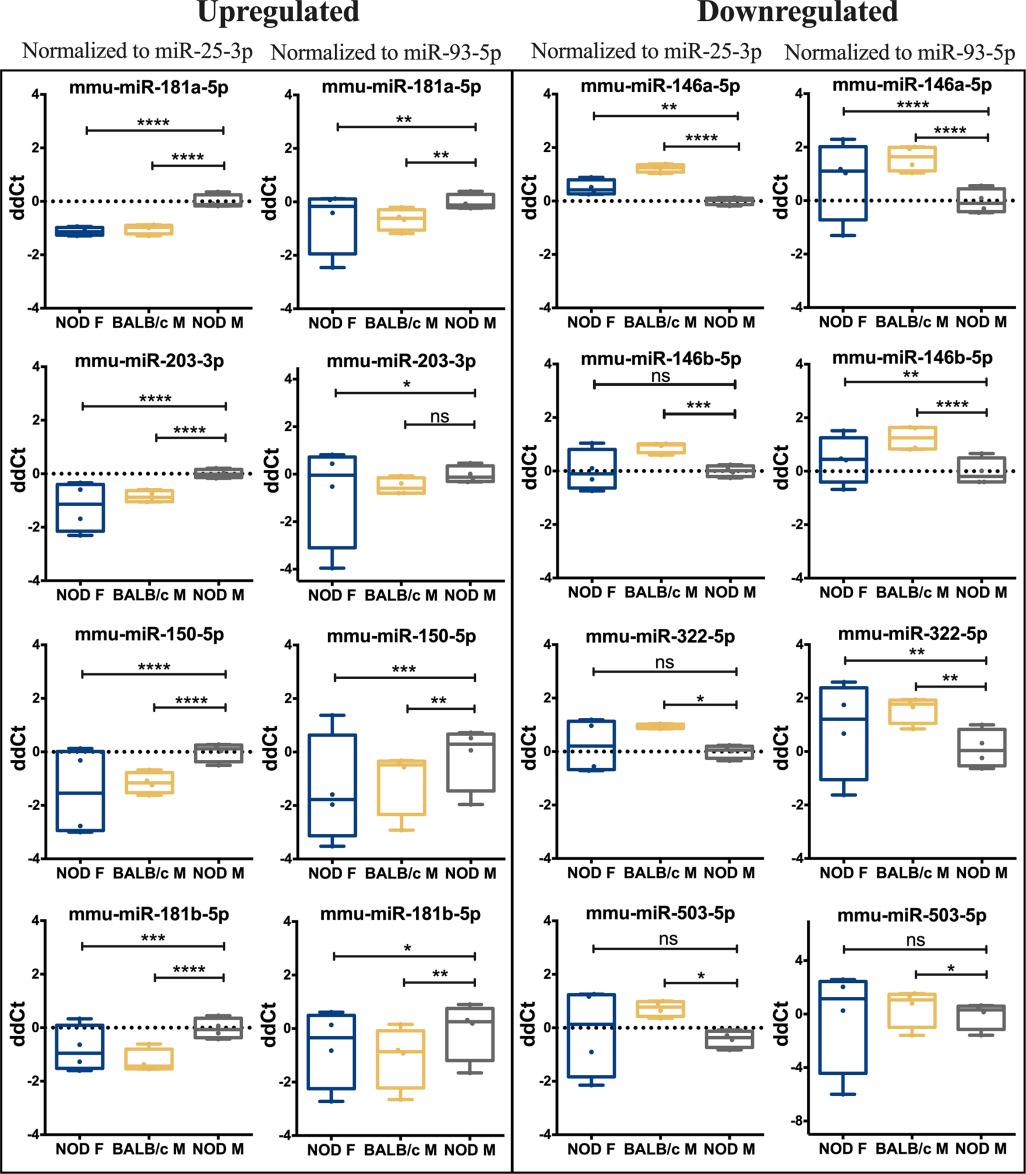
qRT-PCR validation of miRNA hits in mice tears. In a separate cohort of age-matched mice, 13 miRNA hits from the sequencing data set were tested by qPCR to confirm the initial observation. Data was first normalized for plate to plate variation using a spike-in RNA and then to two internal controls, miR-93-3p and miR-25-3p. Of the 13 original hits, 8 miRNA were confirmed as differentially expressed. Statistics were performed on average ΔCt values; data are plotted as ΔΔCt. qPCR was performed with N=4 biological replicate samples, n=3 mice per sample, with 3 technical replicates per sample. (*p < 0.05, **p < 0.01, ***p < 10^-3^, ****p < 10^-4^, Kruskal-Wallis ANOVA in Graph-Pad Prism; ns, not significant).

To validate the observed differential expression of these miRNAs in mouse tears, we collected pooled stimulated tears from additional mouse cohorts for analysis of tear miRNA hits. As with the original discovery cohort, the male NOD mice in this validation cohort had notable autoimmune dacryoadenitis, while the female NOD and male BALB/c mice did not (**Figure 2E**). From the NGS data analysis, we observed that five miRNAs (miR-25-3p, miR-93-5p, miR-16-5p, miR-26a-3p and miR-23a-3p) were unchanged between the three strains (**Supplemental Figures S2A, B**). These miRNAs have been previously identified as endogenous controls in previous miRNA research (50). Additionally, qPCR validation on the same RNA samples showed that miR-93-3p and miR-25-5p were unchanged between the three strains (**Supplemental Figures S2C, D**). Therefore, for our validation study, we used these two miRNAs as endogenous controls, in addition to the spike-in controls UniSp6, and normalized the qPCR data to them in parallel. ‘Hits’ were considered validated if they were significantly different with a fold change in the same direction in male NOD samples compared to both female NOD and male BALB/c samples and when normalized to at least one of the two endogenous miRNA controls. These assays showed that miRNAs miR-146a-5p, miR-146b-5p, miR-322-5p and miR-503-3p were significantly downregulated, while miR-181a-5p, miR-181b-5p, miR-203-3p and miR-150-5p were significantly upregulated in male NOD mice tears as compared to tears of male BALB/c and female NOD (**Figure 5**). However, miR-146b-5p and miR-322-5p were significantly downregulated in both comparisons when normalized to only miR-93-3p, while miR-203-3p was upregulated in both comparisons when normalized to only miR-25-3p (**Table 2**).

Of the hits we could not validate, the miR-147-3p primer exhibited multiple melt curves across all samples for all three replicates and did not meet the quality control requirements for qPCR. miR-3076-3p also did not meet quality control requirements due to failed amplification in 2 out of 4 samples of female NOD and male BALB/c mouse tears, and therefore lacked power for statistical analysis. Differential expression of three other miRNAs, miR-3572-5p, miR-421-3p, miR-3963 could not be validated by qPCR (**Supplemental Figure S3**).

### qPCR Confirms Differential Expression of Some miRNAs in Tears From Patients With SS Relative to Patients With MGD

**Figure 6:**
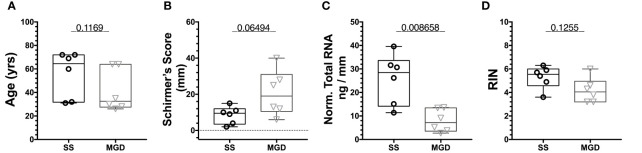
Quality assessment of RNA isolated from patients using Tapestation. **(A)** There was no significant difference in the age of patients from the two groups. **(B)** Schirmer’s test score was higher for SS patients than MGD patients. **(C)** Normalized to tear volume, SS patient tears had significantly higher amounts of total RNA than tears of MGD patients. **(D)** There was no significant difference in the RIN values of RNA from SS and MGD patient tears. N=6 samples each for SS and MGD patients. Samples were analyzed using a Mann-Whitney U test with p<0.05 considered significantly different.

To assess the differential expression of miRNAs in patient tears, we pooled tears acquired from left and right eyes of individual patients with either SS-associated dry eye or MGD diagnosed as described in *Methods*, prior to RNA isolation. The mean age of the two patient groups, SS and MGD, were comparable with no significant differences (**Figure 6A**). We then compared tear volumes as well as concentration and quality of RNA isolated from these tears. As expected, SS patients had lower Schirmer’s test scores relative to patients with MGD (**Figure 6B**). Normalized to mm of wetting of the Schirmer’s strip, the RNA amount (ng) in tears of SS patients was significantly higher than that of MGD patients (**Figure 6C**). We obtained on average 9.21 ng/μL (or 184.2 ng) of RNA from SS patient tears and 7.38 ng/μL (or 147.6 ng) of RNA from MGD patient tears. Tear RNA quality of the two groups was comparable(**Figure 6D**).

8 out of the 13 miRNA hits assayed by qRT-PCR in mouse tears have completely identical nucleotide sequences for human and mice. For validation in patient tears, we used the same custom PCR panels as the mouse validation cohort. The 8 identical miRNA PCR amplified as expected, but we also observed amplification of three murine primers for miRNAs miR-322-3p, miR-503-5p and miR-3963. This is likely due to high sequence similarity of murine microRNAs miR-322-3p and miR-503-5p with their respective human homologs. We observed that miRNA hsa-miR-203a-3p was the most highly expressed of these miRNAs in patient tears, followed by hsa-miR-146a-5p, hsa-miR-150-5p and mmu-miR-3963 (**Supplemental Figure S4**).

**Figure 7:**
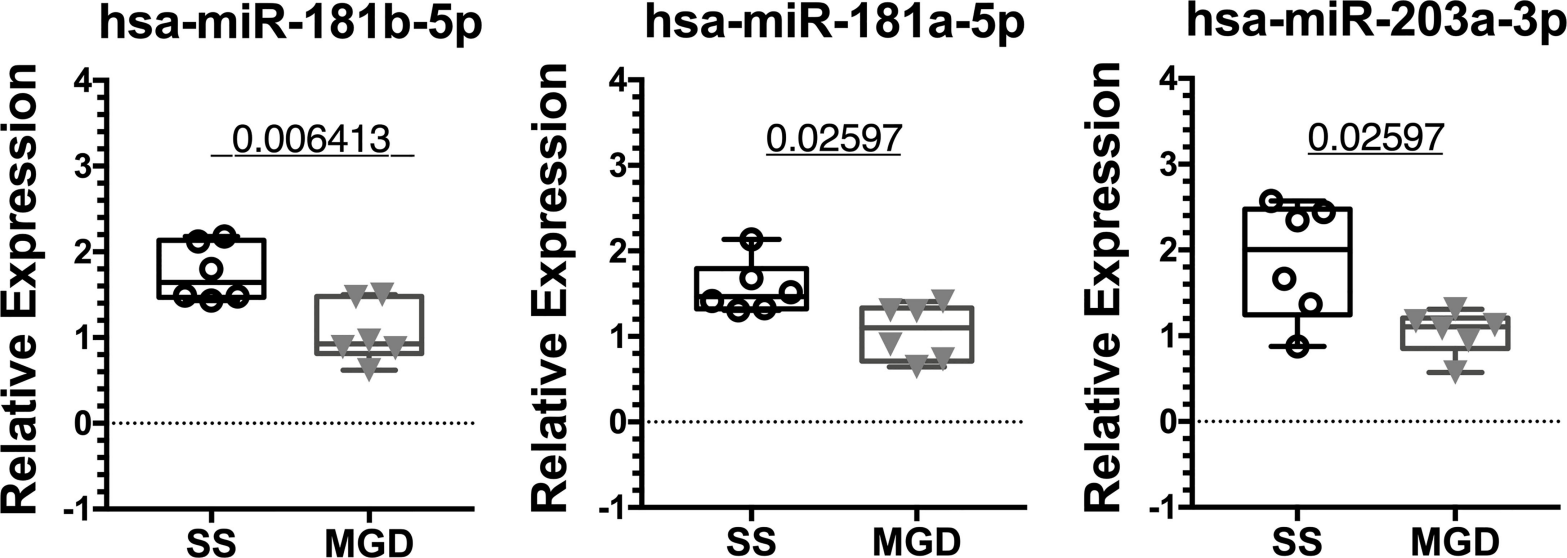
qRT-PCR validation of miRNA hits in human tears from patients with SS or MGD. In a small cohort of patients, miRNAs hsa-miR-181a-5p, hsa-miR-181b-5p and hsa-miR-203a-3p were significantly upregulated in tears of SS patients when compared to tears of patients with MGD. Data were first normalized for variation arising from RT and PCR using spiked-in control. Statistics were performed on ΔCt values normalized to hsa-miR-25-3p. Mean ΔΔCt values are plotted as boxplots showing the mean with 75% to 25% IQR and whiskers depicting the range. (N=6 samples each for SS and MGD patients; unpaired t-test with equal variances assumed; p=0.05).

For qPCR validation, hsa-miR-93-5p and hsa-miR-25-3p were used as endogenous controls for assay normalization and their levels did not vary between the two groups, similar to the observation in male NOD mice tears (**Supplemental Figures S2E, F**). Of the 8 miRNA hits with identical sequences, miRNAs hsa-miR-181a-5p, hsa-miR-181b-5p and hsa-miR-203a-3p were significantly upregulated in tears of patients with SS-like dry eye when compared to tears of patients with MGD when normalized to hsa-miR-25-3p (**Figure 7**).

### IPA Identifies Dysregulation of Molecular Functions and Immunomodulation

**Figure 8:**
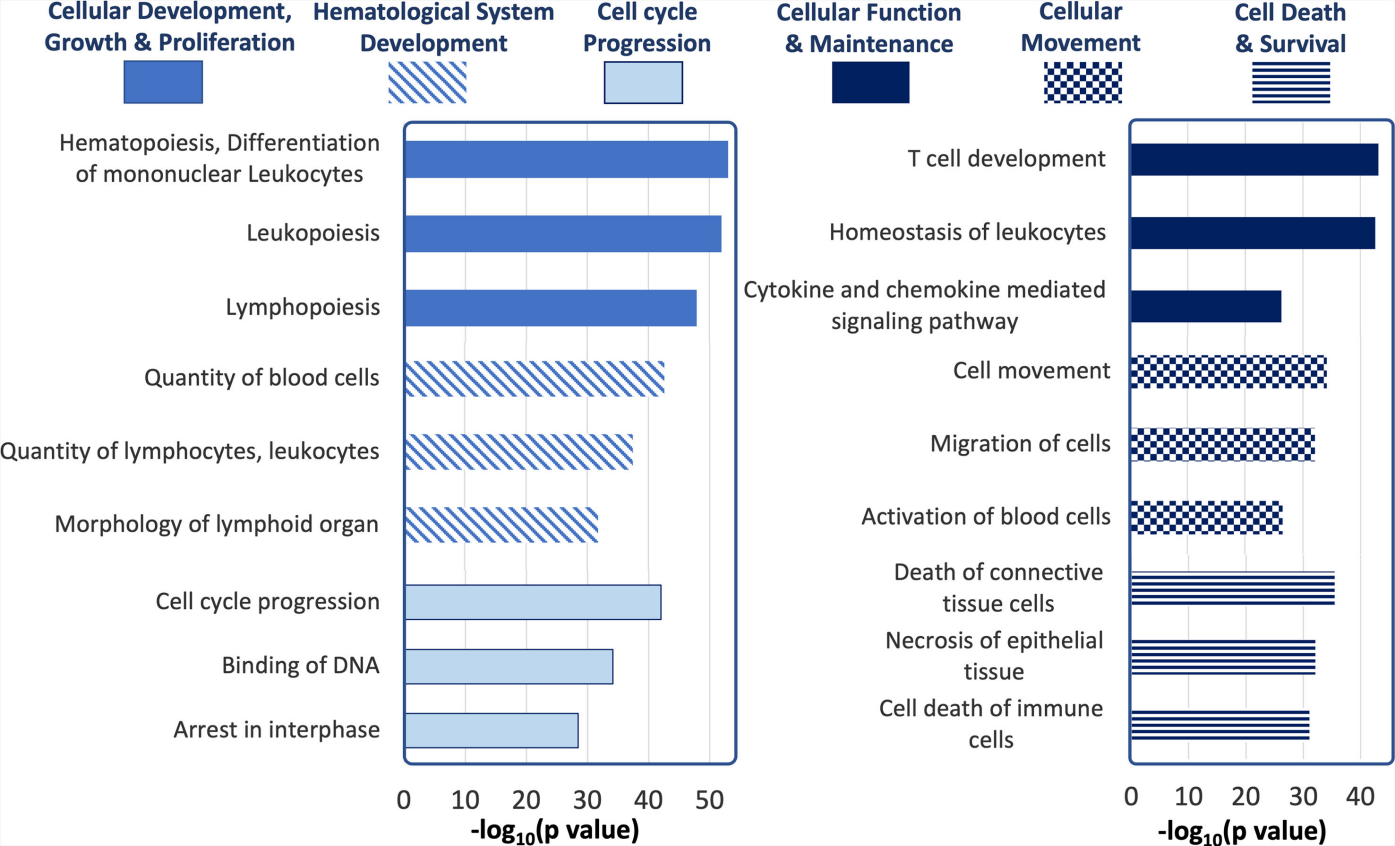
Ingenuity Pathway Analysis of tear miRNA hits. The most statistically significant biological functions and processes generated by IPA for the genes targeted by the differentially expressed tear miRNA hits, grouped by function categories (p values calculated by IPA based on **Table 1**).

To understand the pathways and cellular processes involving the qPCR-validated dysregulated miRNAs, we used Ingenuity Pathway Analysis (IPA). IPA identified mRNA targets of hits that are a) experimentally validated or b) predicted targets with high probability scores (from the TargetScan database). Biological processes with a relatively higher number of such mRNA targets are more likely to be dysregulated by altered expression of miRNA hits and are, therefore, termed ‘overrepresented’. **Figure 8** shows the processes most likely to be affected, in decreasing order of statistical probability. The most overrepresented cellular processes and functions were differentiation and hematopoiesis of mononuclear leukocytes (p<10^-55^), leukopoiesis (p<10^-53^) and lymphopoiesis (p<10^-48^), followed by proliferation of connective tissue cells (p <10^-42^), lymphatic system cells (p<10^-42^) proliferation of, mononuclear leukocytes (p<10^-41^) and lymphocytes (p <10^-41^). Proliferation processes related to other cell types, such as those of liver (p<10^-16^), kidney (p<10^-12^), and heart (p<10^-6^) were relatively underrepresented. Among cellular processes leading to disease development, cancers such as nonhematologic malignant neoplasm and head and neck tumor was the most over-represented (p<10^-43^).

**Figure 9:**
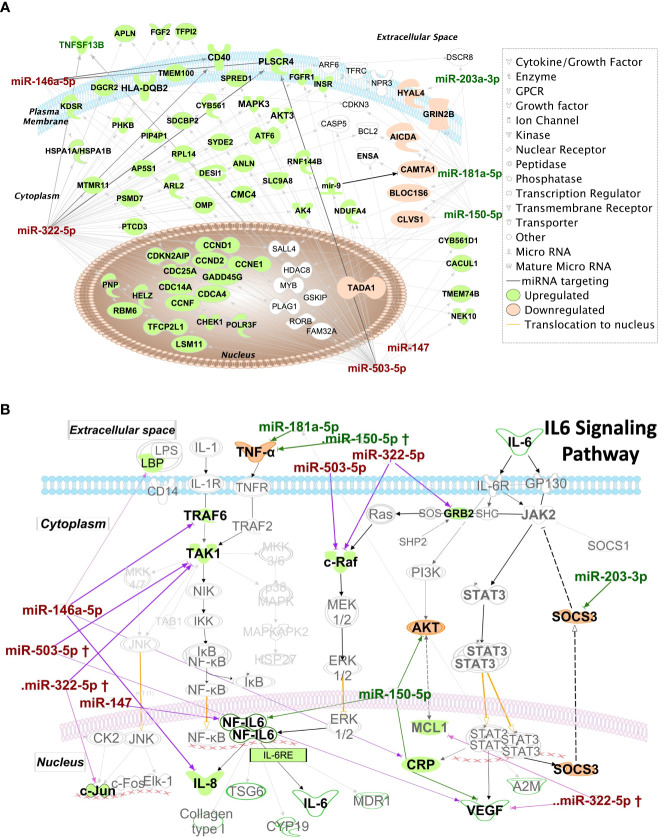
IPA Network analysis of targeted miRNAs. **(A)** IPA Network map of miRNA hits and their mRNA targets. Genes targeted by >1 miRNA hits are shown according to their subcellular localization. Arrows represent post-transcriptional silencing of genes by connected miRNA. Downregulated miRNA hits are shown in red, upregulated ones are in green, whereas gene targets likely to be upregulated are shaded in green, and those likely to be downregulated are shaded in orange. Gene icons shaded in white are targeted by both up and downregulated miRNA. **(B)** Pathway analysis results suggest upregulation of IL-6 family cytokines and those transcribed through the IL-6-response element (IL6RE). In the NF-κB signaling, TRAF6 and TAK1 are targets of hits miR-146a-5p and miR-322-5p. Downregulation of these hits may lead to upregulation of TRAF6 and TAK1 and increase the transcription of the IL6RE, resulting in increased mRNA levels of cytokines. Of these, IL-8 is directly targeted by miR-146a-5p and its levels could be particularly upregulated with depletion of this miRNA. Also of key import is the targeting of SOCS3 by the upregulated miRNA miR-203-3p. SOCS3 is a negative regulator of the JAK2/STAT3 pathway and is required to turn off the pathway to prevent excessive production of cytokines.

**Figure 9A** shows mRNAs targeted by more than one miRNA hit, shown within the context of the subcellular location of the encoded protein. The mRNA targets include those predicted to be targeted by miRNA hits with high probability based on in-silico seed region matching, and targets that have been experimentally validated. Several mRNA targets appear to be transcription factors implicated in cell cycle progression such as CDKN2AIP, CDC25A, CDCA4, CDC14, CCDN1 and CCDN2. Also, of interest is PLSCR4 (Calcium dependent Phospholipid scramblase 4), an ATP-independent, transmembrane lipid transporter responsible for inducing non-specific bidirectional movement of phospholipids in the cell membrane during cell activation that has also been proposed as a potential mediator of phosphatidylserine in the outer leaflet of the plasma membrane in apoptosis (51). PLSCR4 is targeted by three of the downregulated miRNA hits, miR-146a-5p, miR-322-5p and miR-503-5p. Genes targeted by miRNA hits known to be involved in immunological processes include CD40, TNFSF13B and HLA-DBQ2, with their expression predicted to increase due to the observed downregulation of their regulating miRNAs. Interaction of CD40 with its ligand CD40L (or CD154) plays an important role in immunity as it promotes plasma cell differentiation, antibody production and is required for optimal immune response to most antigens. This is in line with previous studies demonstrating that CD40 is overexpressed in SG ductal epithelial cells, lymphocytes, and endothelial cells in SS patients (52). Antibody blockade of CD40 has also shown therapeutic potential for treatment of SS in clinical trials (53).

Several key proinflammatory mediators are also targets of dysregulated miRNA. To illustrate this, IPA results focused solely on IL-6 signaling pathways are shown in **Figure 9B**. IL-8 and C-reactive protein (CRP) mRNAs are targets of the downregulated miRNA hits, miR-322-3p and miR-146a-5p. TAK1 and TRAF6 of the NF-kB pathway and c-Raf of the MAPK/ERK pathway are also targets of the downregulated hits, miR-322-5p, miR-146a-5p and miR-503-5p. This downregulation may lead to upregulation of IL-6-Response elements (**Figure 9B**) promoting upregulation of cytokines such as IL-6 and IL-8 which are highly relevant to SS pathogenesis and upregulated in SG (54), serum and tears (55) of SS patients. SOCS3, a negative regulator of the JAK2/STAT3 pathway, is targeted by overexpressed miR-203-3p, which may result in its downregulation leading to the constitutive transcription of CRP, VEGF and A2M (**Figure 9B**). SOCS3 downregulation may lead to enhanced activity of pro-inflammatory IL6 family cytokines through its reduced ability to interact with gp130 (56) to modulate IL6 signaling, also potentially contributing to SS pathogenesis.

## Discussion

Dry eye disease is a multifactorial condition linked to chronic inflammatory diseases, environmental challenges, hormonal status and even medication, However, SS-associated dry eye, which occurs through a distinct mechanism involving development of autoimmune inflammation and loss of secretory function of the LG, is arguably the most debilitating. In the absence of a definitive diagnosis and without clinical intervention, SS patients with dry eye symptoms can develop corneal lesions which can require corneal transplantation to restore vision (57, 58). Unchecked autoimmune inflammation in exocrine tissues including LG can lead to formation of ectopic germinal centers which may also contribute to autoantibody production and other mediators of extraglandular symptoms of SS (59, 60). Clinical diagnosis of dry eye largely evaluates functional measures such as tear flow, tear osmolarity, tear break up time and ocular surface integrity; there are no diagnostic tests to directly assess inflammation of the LG. While the LG cannot be biopsied owing to the substantial risk of damage, tear analysis is feasible. Soluble constituents of tears are produced and secreted largely by the LG; thus, any damage, immune infiltration, deposition, or fibrosis in the LG should be reflected by changes in the tear composition. Tear analysis thus provides critical insights into disease processes ongoing in the LG at different stages.

Although symptoms of dry eye disease are a part of the ACR-EULAR criteria for SS diagnosis, there are no specific tests which can easily detect autoimmune involvement of the LG relative to the use of the minor SG biopsy which accompanies identification of oral dryness to aid in diagnosis of SS with SG involvement. SS patients report uneven manifestation of ocular versus oral symptoms (61), suggesting that exocrinopathies of the LG and SG may not always develop in parallel. Murine models of SS also manifest different patterns of LG and SG exocrinopathy (31, 62). The availability of a test that could enable eye care specialists and others to distinguish patients with SS-associated dry eye disease versus those with dry eye due to other causes, could improve diagnosis for the 15% of SS patients who present primarily with dry eye symptoms (63). One study has shown that the diagnosis of SS patients presenting with only dry eye symptoms is 2.7 times more likely to be delayed (63). More patients presenting with only dry mouth symptoms (53%) are accurately diagnosed with SS within a year, relative to the accuracy of diagnosis of patients presenting with dry eye symptoms within the same time frame (41%). About 42% of patients with only dry eye symptoms take >1 year to be diagnosed accurately (63) putting patients with a more prominent ocular symptoms at a disadvantage. As an additional benefit, tears can be collected non-invasively using Schirmer’s strips (64), obtained as part of a common clinical test for dry eye. Dysregulated miRNAs can be measured at any time after tear collections are frozen and can be multiplexed to detect multiple species, as they are chemically stable when stored at -80°C (65).

Tears are a viable source for identification of unique miRNAs denoting disease. Previous studies comparing various biofluids have reported that human tears contain over 600 distinct proteins (66) and over 300 miRNAs (17). Tears also have a higher concentration of miRNA than does urine, CSF or plasma (17). Tear miRNAs are thought to play important roles in maintaining the health of the ocular surface and mediating immune response during infection. Recently, tears have been investigated for miRNA biomarkers for Alzheimer’s diseases (15) and primary open-angle glaucoma (18) with the identification of hits showing very high sensitivity and specificity (AUC = 0.93) (18). These findings collectively suggest that changes in the tear miRNAome can reflect disease status. To our knowledge, the tear miRNAome in SS has not been comprehensively sequenced.

Our study has identified >550 distinct miRNAs in NOD mouse tears, substantially more than reports from a previous study investigating 43 tear miRNAs using qPCR (16). Our approach utilized NGS, which provides a broader, more comprehensive, and unbiased genome readout of RNAs than do microarrays. Only 2 of our 8 validated miRNA hits, mmu-miR-146a/b-5p, have previously been linked to ocular symptoms of SS while 4 of 8 of our validated miRNAs hits including miR-181b-5p, miR-203-3p, miR-322-3p and miR-503-5p have never been previously linked to SS. Two of these - miR-181b-5p, miR-203-3p, were validated in the small pilot study of 6 SS patients in **Figure 7**. Nearly 70% of human miRNA have mouse orthologs with conserved seed-region sequences (67). In our study, 6 of the 8 validated miRNA hits– mmu-miR-146a-5p, mmu-miR-146b-5p, mmu-miR-150-5p, mmu-miR-181a-5p, mmu-miR-181b-5p, and mmu-miR-203-3p – are completely identical between human and mouse (67). This highlights the utility of miRNA research in murine models to study development of SS as we have demonstrated in our pilot study. Of the identified miRNAs that are not completely identical between human and mouse, miR-503-5p differs from its human counterpart (hsa-miR-503-5p) by 1 nucleotide while identified orthologs are known for miR-322-5p (hsa-miR-424-5p), so both miRNAs could likewise be evaluated in any analyses of SS patient tears or tissue.

miR-150-5p, miR-146a/b-5p, and miR-181a-5p, identified here as dysregulated in NOD mouse tears, and hsa-miR-181a-5p, in SS patients, have been previously linked to SS in other studies. One study investigating peripheral blood mononuclear cells (PBMC) from SS, SLE and healthy control subjects found miR-150-5p to be the only miRNA downregulated in SS patients as compared to SLE and healthy controls (68). However, other studies have found miR-150-5p to be elevated in SS patients’ saliva, minor SG (69), and serum (70) (**Table 3**), consistent with the increased expression in male NOD mice tears. miR-150-5p was previously found to be significantly upregulated in a rabbit autoimmune dry eye model investigating differential expression of miRNAs in LG (71). This may be related to the observed upregulation of miR-150-5p in NOD mouse tears, as the LG is the primary source of tears. We also analyzed preexisting raw datasets generated by other groups (72) (SRA Accession: PRJNA542600, ENA Accession: PRJDB9749) in mouse models of SS that were made publicly available at Sequence Read Archive (SRA) and European Nucleotide Archive (ENA). In LG of *Aire* knockout mice, precursor miRNA mir-503 was 4-fold downregulated as compared to LG of wild type mice. In LG of male NOD mice, gene expression of precursor miR-181a was 3-fold upregulated as compared to male BALB/c. This is similar to what we observe for mature miRNAs miR-503-5p and miR-181a-5p in tears of the NOD model.

**Table 3:** Literature summary for previous miRNA hits in studies on SS.

microRNA	Immune Cells	Salivary Gland	Serum	Tears
**miR-146a-5p**	PBMC (68, 73–76)↑	Mouse SG (75)↑		Tears (16) ↑
	T↑ & B↓ Lymphocytes (77)			
**miR-146b-5p**	PBMC (68, 73)↑	SG (69)↑	–	Tears (16) ↑
**miR-181a-5p**	PBMC (68, 76, 78)↑	SG (79)↓	–	–
		SG, SG Epithelial Cells (78)||, *SG (69)↑		
**miR-150-5p**	PBMC (68, 80)↓	SG (69)↑	Serum (70)↑	–
	Naïve B cell (70, 81) ↑			
	Activated B cell (81) ↓			

miRNA in bold have identical sequence for human and mouse; *Upregulated in SS patient SG with decreased salivary flow; ↑ upregulated; ↓ downregulated; || no change; PBMC, Peripheral mononuclear blood cells.

Altered expression of miR-146a-5p and miR-146b-5p has been reported in plasma/serum and PBMCs of SS patients as well as in saliva, SG and LG from animal models of SS (**Table 3**). A microarray of 43 miRNAs in SS patient tears showed that miR-146a-5p was downregulated in tears of primary SS patients [i.e., SS patients who lack other autoimmune diseases (1)] relative to healthy subjects but was significantly upregulated in tears of patients with secondary SS (i.e., SS patients who also have other autoimmune diseases (1)) relative to patients with primary SS (16). In SS-prone B6DC mice, miR-146a levels were upregulated in LG and submandibular SG at 8 weeks but downregulated at 20 weeks, while its levels were increased significantly in PBMC of 20 weeks old mice (75). Dysregulated expression of miR-146a/b-5p has also been found in animal studies and in various biofluids in patients with SLE and RA and may serve as a more general indicator of autoimmunity. A more comprehensive analysis of the changes in miR-146a/b-5p over time in tears, in parallel with a more comprehensive analysis of LG disease status, may be necessary to understand its utility in diagnosis of SS.

Dysregulation of miR-181a-5p has also been described in SS, with several studies reporting its upregulation in PBMC of SS patients relative to healthy subjects (**Table 3**). We found it to be upregulated in tear of patients with SS-like dry eye in our pilot study compared to those of patients with MGD. In SG of SS patients, this miRNA was downregulated relative to healthy controls, but within the SS patient cohort it was upregulated in the SG of those exhibiting more profound decreases in salivary flow and high SG focus scores (69). Intriguingly, this miRNA is also implicated in regulation of the differentiation of germinal center B cells (82), which may also be found in the ectopic germinal centers that form at late stages of SS in exocrine glands.

All validated and dysregulated miRNAs identified here are expressed in blood cells and function in immune cell development and differentiation. Unsurprisingly, pathway analysis of the miRNA hits identifying likely gene targets and the biological processes involving these gene products include differentiation, maturation and proliferation of T and B lymphocytes. Tears produced by the LG and other ocular surface tissues drain through the canaliculus into nasolacrimal ducts. Here, tear components including miRNAs can be reabsorbed into the blood vessels surrounding the cavernous body of the nasolacrimal ducts (19) and shuttled back to the LG. These tear constituents may have access to draining lymph nodes through the tear duct associated lymphoid tissue (TALT). We hypothesize that a primary function of tear miRNAs is the regulation and homeostasis of local immune responses in the LG and ocular surface; dysregulated miRNAs may therefore contribute to induction and perpetuation of autoimmune processes by priming immune cells in the TALT that migrate to the LG in SS.

Precursors mir-146a/b and mir-181b miRNAs participate in immune cell development and differentiation of T and B cells from hematopoietic stem cells (83, 84). miR-150-5p is decreased in serum of patients with B cell malignancies, and in double negative thymocytes, but its levels increase in differentiating T lymphocytes (84, 85). miR-150-5p is highly expressed in naïve T and B cells, with levels decreasing in mature B cells (85). miR-150-5p is thought to maintain B cells in the quiescent stage in lymphoid organs and to regulate their expansion by targeting the transcription factor MYB (86). It is also involved in natural killer cell maturation and development (87). These findings, along with our observation of its upregulation in male NOD tears, suggest it may participate in local T and B cell maturation with its levels possibly reflecting the degree of immune cell infiltration of the LG.

Murine miR-322 promotes Th17 cell differentiation through its targeting of the transcription factor NF-kB subunit p50 (88), which is interesting because of the prominent role of Th17 and IL-17 as disease drivers in SS (89). miR-322 may also act as an anti-inflammatory agent through its ability to suppress cytokine secretion and signaling (90). This function is consistent with our IPA analysis which highlights the targeting of master regulators of IL-6 production (TAK1 and c-Raf) by miR-322-5p (**Figure 9B**). Consistent with this, we have previously reported that IL-6 gene expression increases significantly in LG of male NOD mice following the onset of autoimmune dacryoadenitis, as compared to LG of age-matched male BALB/c and female NOD mice (20). hsa-miR-424, the human ortholog of murine miR-322, as well as miR-503-5p are also implicated in monocyte differentiation (91, 92). Suppressor of cytokine signaling 3 (SOCS3), a negative feedback regulator of the JAK2/STAT3 pathway, is targeted by upregulated miR-203-3p which may lead to its decreased expression. As a result, IL6-mediated signaling of the JAK2/STAT3 pathway may be enhanced in cells with increased miR-203-3p.

One limitation of our study is our use of only a single model of SS. As well, SS patients are predominantly female (93), yet the male NOD mouse is one of the most common murine models used to evaluate SS-associated dry eye disease. Use of the male NOD mouse in our hands has led to discovery of the tear biomarker, cathepsin S, which has been validated as selectively and highly upregulated in two separate cohorts of female SS patients (27, 94). Exploration of the role of sex hormones in development of autoimmune dacryoadenitis has shown that LG inflammation in this model is induced by male sex hormones; orchiectomized mice exhibit a lesser immune infiltration into LG (31). Moreover, adoptive transfer experiments of splenocytes between male and female NOD and NOD SCID mice confirm that the lacrimal gland microenvironment in the male NOD mouse contributes uniquely to lymphocytic infiltration (31, 95). Male sex hormones do not have a comparable effect in another autoimmune model of SS and other autoimmune diseases, the MRL/lpr mouse (62). Intriguingly, ductal epithelial cell from labial salivary gland biopsies in SS patients were positive for testosterone, dihydrotestosterone and estradiol, whereas cells from healthy control biopsies were positive only for estradiol (96). Thus, much remains unknown regarding the interaction between sex hormones and LG tissue in disease.

The male and female NOD mice are also predisposed to development of diabetes, although at an older age than the 12–14-week aged mice used for tear collection. Thus, this predisposition represents a confounding factor. Animal models may also not fully represent the complexity of SS disease progression. In addition, the pooling strategy used to obtain RNA of sufficient quantity and quality may have reduced our overall sensitivity, especially for miRNAs that are lowly expressed. However, these conditions were still sufficient to detect several significantly altered miRNAs, half of which were experimentally validated in separate samples. Moreover, 3 of these miRNAs were validated in a small pilot study of SS patients. The size of our human cohort being extremely small is also a limitation, and future studies will require investigations with much larger cohorts and additional control groups.

In conclusion, we report here the identification and validation of 8 dysregulated miRNAs in tears that have potential for use in diagnosis of ocular (LG) involvement in SS. Of these, 4 have been previously identified in other biofluids or organs in SS patients or disease models and we have validated 1 in a pilot study in SS patient tears; while 4 are newly implicated in SS of which we have validated 2 in our pilot study with SS patient tears. This list of differentially expressed tear miRNAs is first of its kind, is of high priority for future investigation in SS patient tears and may potentially distinguish ocular involvement in SS patients.

## Data Availability Statement

The data used to support the findings of this study are included within the article and **Supplementary Materials**. The raw datasets generated as part of this study can be found in the Sequence Research Archive under BioProject Accession ID PRJNA769738 and are available for download here https://www.ncbi.nlm.nih.gov/Traces/study/?acc=PRJNA769738&o=acc_s%3Aa. The code used for analysis of the generated datasets can be found here https://github.com/singhkakan/miRGrep.

## Ethics Statement

The animal study was reviewed and approved by The Institutional Animal Care and Use Committee (IACUC). All procedures in the human subjects study were approved by the USC Institutional Review Board and were performed in accordance with the Declaration of Helsinki, and all subjects provided written informed consent. Use of samples from the USC Dry Eye Center of Excellence tear biorepository at the USC Roski Eye Institute tear biorepository is approved under IRB protocol HS-19-01004 and meets the requirements for ‘nonhuman research with biospecimens previously generated/collected to be analyzed for research purposes (Data and Specimens) or secondary data analyses.

## Author Contributions

All authors were involved in drafting the article or revising it critically for important intellectual content, and all authors approved the final version to be published. SK wrote the first draft. BH, SH-A, SK, and ME were involved in the study conception and design. SK, AY, and AN acquired the data. SK, BH, SH-A, ME, CO, and AN were involved in the analysis and interpretation of data.

## Funding

This work was supported by RO1 EY011386 to SH-A. Further support for the project came from P30EY029220, and an unrestricted departmental grant from Research to Prevent Blindness (RPB), New York, NY 10022.

## Conflict of Interest

The authors declare that the research was conducted in the absence of any commercial or financial relationships that could be construed as a potential conflict of interest.

## Publisher’s Note

All claims expressed in this article are solely those of the authors and do not necessarily represent those of their affiliated organizations, or those of the publisher, the editors and the reviewers. Any product that may be evaluated in this article, or claim that may be made by its manufacturer, is not guaranteed or endorsed by the publisher.
