# Supplementary material for: Tear miRNAs Identified in a Murine Model of Sjögren’s Syndrome as Potential Diagnostic Biomarkers and Indicators of Disease Mechanism
Source: Front Immunol. 2022 Mar 4;13:833254. doi: 10.3389/fimmu.2022.833254 (PMC8931289; doi:10.3389/fimmu.2022.833254)
Supplement: Supplementary file 1 [file DataSheet_1.pdf]

Tear miRNAs identified in a murine model of Sjögren's Syndrome as potential diagnostic biomarkers and indicators of disease mechanism

Shruti Singh Kakan[1,2], Maria C. Edman[2], Alexander Yao[2], Curtis T. Okamoto[1], Annie Ngyuen[2],

Brooke E. Hjelm[3*], and Sarah F. Hamm-Alvarez[1,2*]

[1]Department of Pharmacology and Pharmaceutical Sciences, School of Pharmacy, University of Southern California, Los Angeles, CA, United States

[2]Department of Ophthalmology, Roski Eye Institute, Keck School of Medicine, University of Southern California, Los Angeles, CA, United States

[3]Department of Translational Genomics, Keck School of Medicine, University of Southern California, Los Angeles, CA, United States

***Co-corresponding authors**
Address correspondence to:

Sarah F. Hamm-Alvarez
shalvar@usc.edu

Brooke E. Hjelm
bhjelm@usc.edu

Keywords: miRNA, NOD mouse, autoimmune disease, tears, Sjögren's syndrome, Next-generation sequencing

1. Supplemental Figures and Tables

1.1 Supplemental Figures

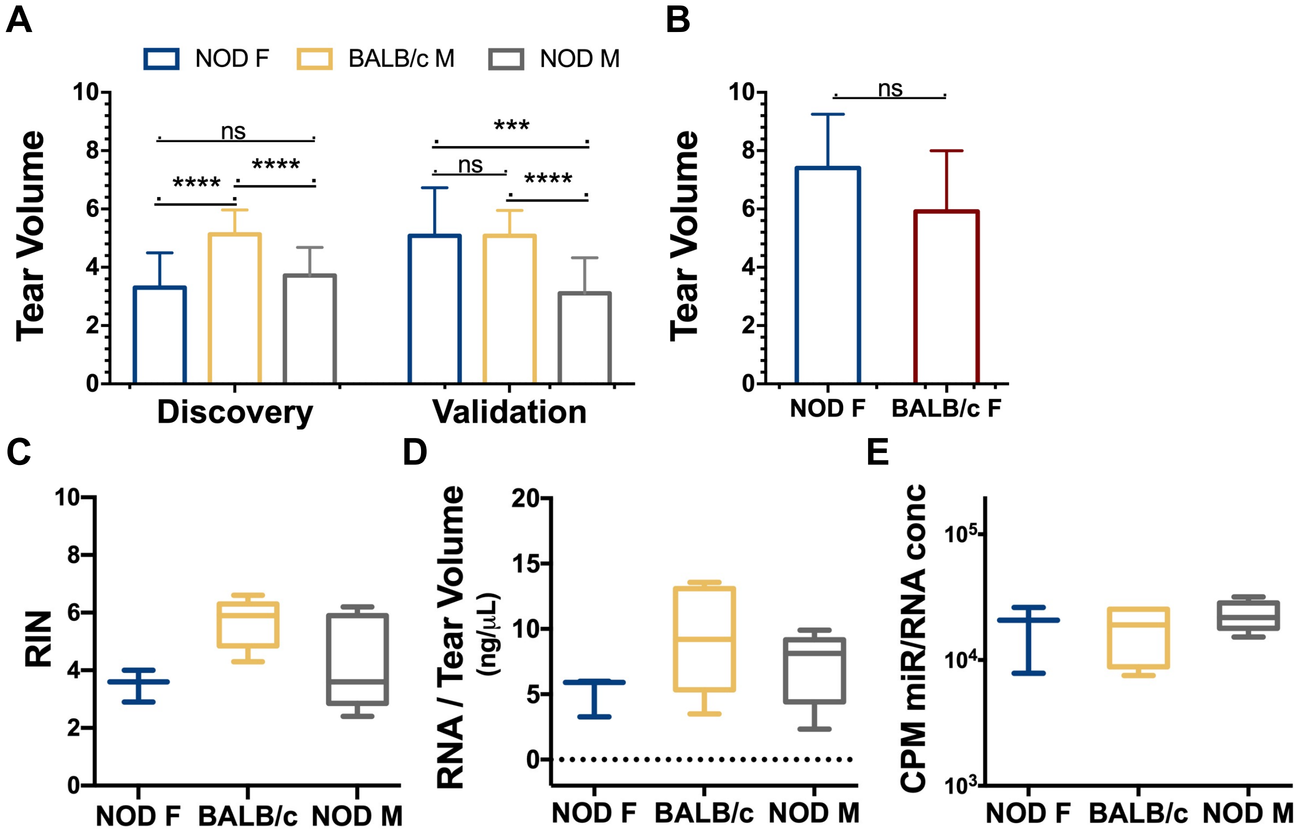

Figure S1. Tear volumes and quality assessment of RNA isolated from pooled tears of 12-14 weeks old female NOD, male BALB/c and male NOD mice using TapeStation. (A) Tear production in male NOD mice was significantly lower as compared to male BALB/c mice in both discovery and validation cohorts whereas it was significantly lower than the female mice in the validation cohort only. (* $p < 0.05$, ** $p < 0.01$, *** $p < 10^{-3}$, **** $p < 10^{-4}$, ns – not significant, 2-way ANOVA with Tukey's test for correction for multiple comparisons, at $p < 0.05$). (B) Comparison of tear volumes of 14-week-old female NOD and female BALB/c mice showing no significant change (mice were not part of discovery or validation cohorts). (C) There was no significant difference in the amount of RNA isolated relative to the total tear volume isolated for each sample. (D) There was no significant difference in the RNA Integrity Number (RIN) values between samples from the three groups. (E) Counts per million (CPM) miRNA reads for each sample aligning to miRbase v22.0 did not differ significantly between the three groups. Data are plotted as boxplots showing mean with 75% to 25% IQR and whiskers show the range. N=5 samples for male NOD and BALB/c, 3 samples for female NOD; n=5 mice per sample. Samples analyzed by Kruskal-Wallis ANOVA with p<0.05 considered significantly different.

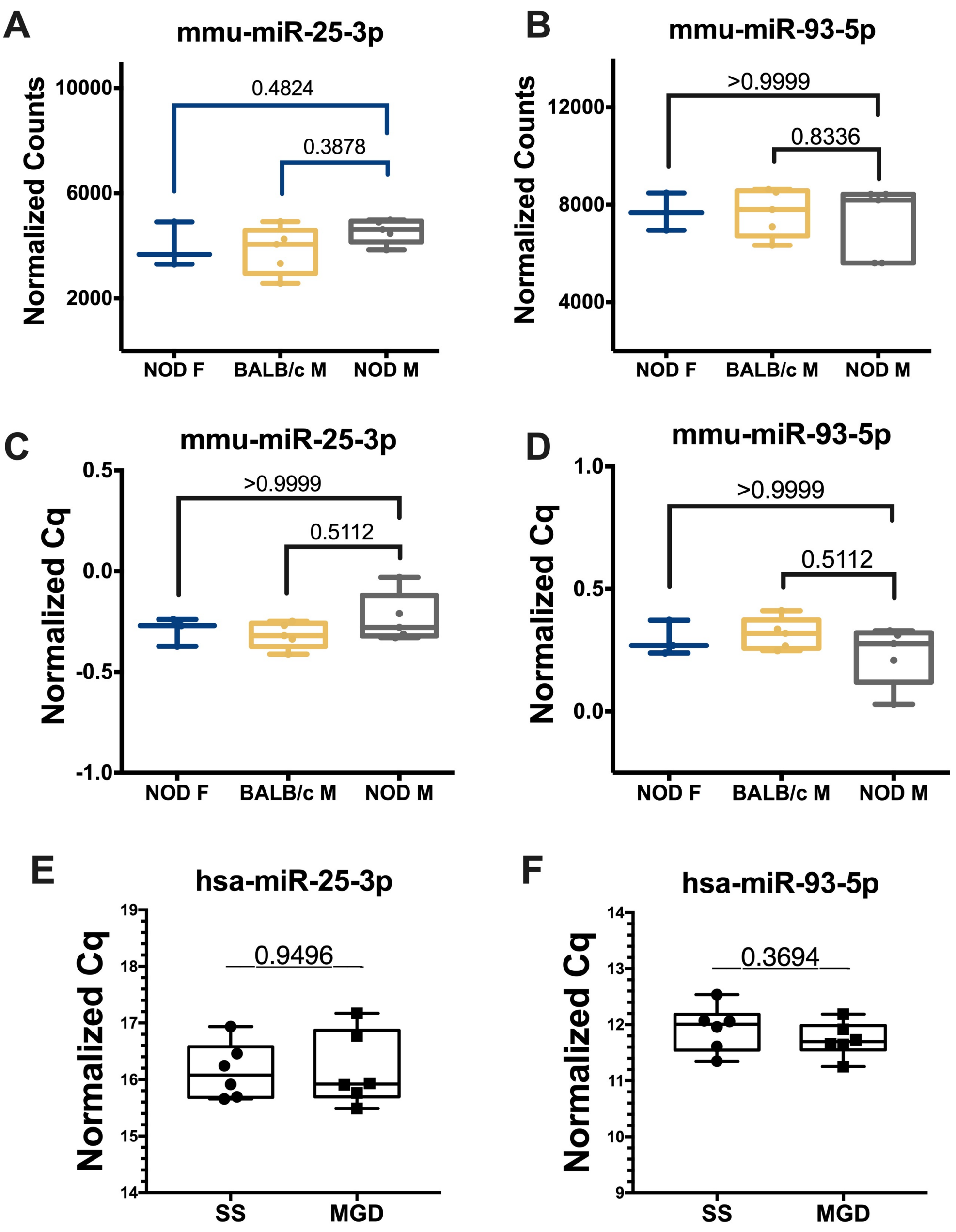

Figure S2. Comparison of expression of endogenous controls miRNAs mmu-miR-25-3p and mmu-miR-93-5p in RNA isolated from tears of male NOD, female NOD, male BALB/c mice and human tears. miRNA sequencing data analysis of mouse tears showed that there were no significant differences in the expression levels of (A) miR-25-3p and (B) miR-93-5p. Data for (A) and (B) are counts normalized by DESeq2 plotted as boxplots showing all points from minimum to maximum. N=5 samples for male NOD and BALB/c, 3 samples for female NOD; n=5 mice per sample. On the same set of samples, qPCR showed that miRNAs (C) miR-25-3p and (D) miR-93-5p had very similar expression levels between the three strains. Median Cq values are plotted as boxplots showing mean with 75% to 25% IQR and whiskers show the range. N=5 samples for male NOD and BALB/c, 3 samples for female NOD; n=5 mice per sample, Kruskal-Wallis ANOVA, p=0.05. With human tear RNA samples from SS and MGD patients, qPCR showed that miRNAs (E) miR-25-3p and (F) miR-93-5p had no difference in expression levels. N=6 samples for SS and MGD. Unpaired t-test with equal variances assumed; p=0.05.

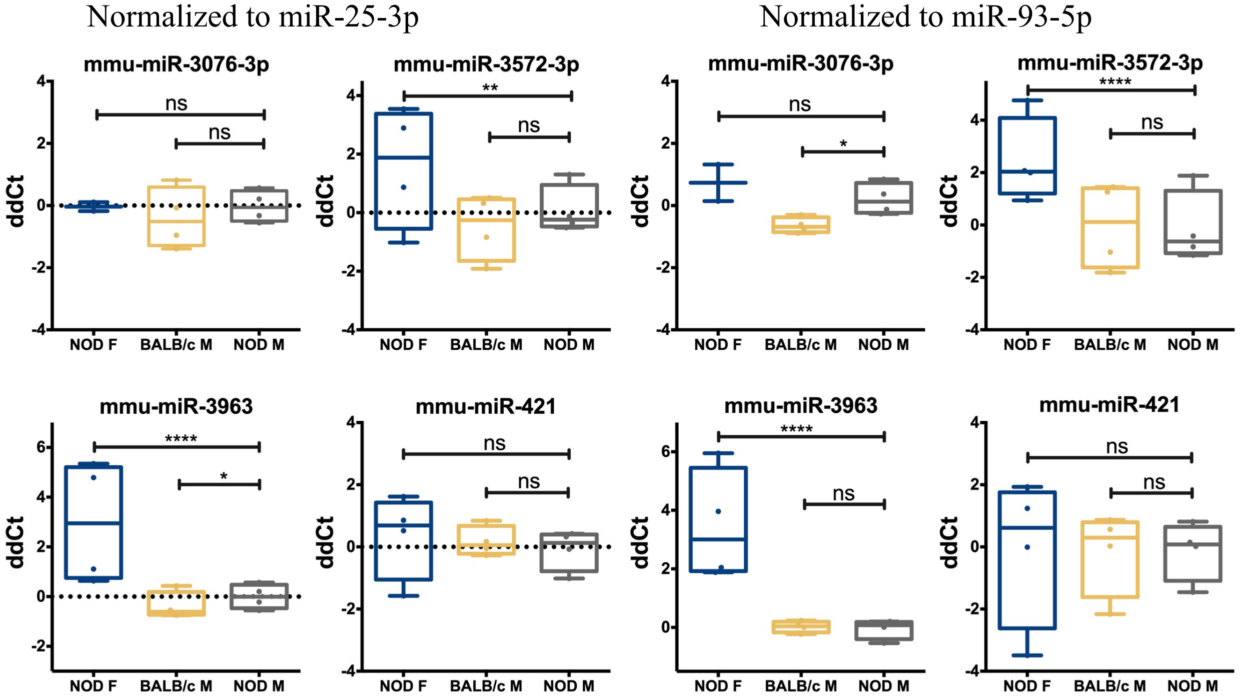

Figure S3. Assessment of miRNA hits predicted from the bioinformatics analysis which were not validated by qRT-PCR. Differential expression of 4 miRNAs were not confirmed in the qRT-PCR validation. miRNA miR-3076-3p expression was not significantly different from that of female NOD mice and was modestly upregulated in male NOD tears as compared to male BALB/c. miR-3572-3p was found to be downregulated in male NOD mice tears as compared to female NODs, but modestly upregulated when compared to male BALB/c. miR-421-3p was modestly downregulated in male NOD tears with respect to male BALB/c and female NOD, but this difference was not significantly different in either comparison. Mean ΔΔCt values are plotted as boxplots showing mean with 75% to 25% IQR and whiskers show the range. N=4 samples for male NOD and BALB/c, and female NOD; n=3 mice per sample. * $p < 0.05$, ** $p < 0.01$, *** $p < 10^{-3}$, **** $p < 10^{-4}$, ns – not significant, Kruskal-Wallis ANOVA, p=0.05.

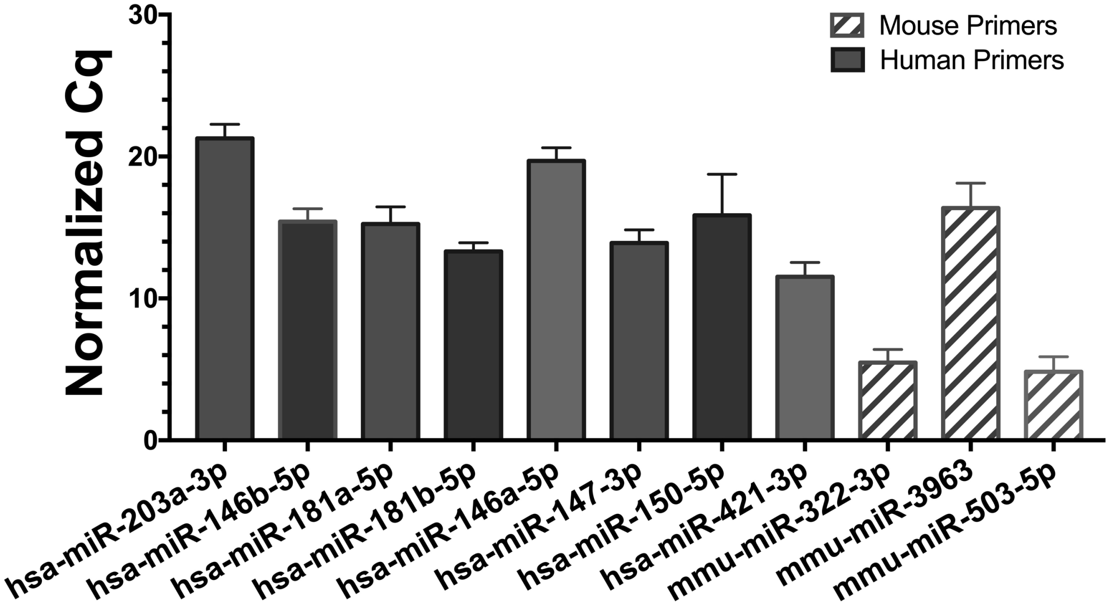

Figure S4. Relative expression of miRNA hits in RNA isolated from patients tears. Primers for 8 miRNAs that have identical sequences for human and mouse (and can be used interchangeably) amplified successfully during qPCR, with hsa-miR-203a-3p having the highest level of expression followed by hsa-miR-146a-5p. 3 of the five mouse specific primers also appeared to amplify successfully during qPCR, with mmu-miR-3963 being expressed at a much higher rate. Data are shown as bar plots of qPCR normalized mean Ct values with error bars showing range (N=12).
